# Genome Sequence of Aspergillus aculeatinus IC_8, Isolated from an Indoor Air Sample of an Urban Housing Complex in Abidjan, Ivory Coast

**DOI:** 10.1128/MRA.00096-21

**Published:** 2021-03-11

**Authors:** Shu Zhao, David Koffi, Jean-Paul Latge, Karidia Sylla, John G. Gibbons

**Affiliations:** a Department of Food Science, University of Massachusetts, Amherst, Massachusetts, USA; b Molecular and Cellular Biology Graduate Program, University of Massachusetts, Amherst, Massachusetts, USA; c Parasitology and Mycology Department, Institut Pasteur de Côte d’Ivoire, Abidjan, Ivory Coast; d Aspergillus Unit, Institut Pasteur, Paris, France; e Organismic & Evolutionary Biology Graduate Program, University of Massachusetts, Amherst, Massachusetts, USA; Vanderbilt University

## Abstract

Aspergillus aculeatinus is an industrially important species of *Aspergillus* section *Nigri* capable of producing bioactive, antibiotic, and antitumor compounds. We sequenced the genome of a strain of *A. aculeatinus* that was isolated from the interior of a housing complex in Abidjan, Ivory Coast.

## ANNOUNCEMENT

*Aspergillus* section *Nigri* (the black aspergilli) consists of species that cause food spoilage, cause plant disease, and produce industrially relevant compounds like lipases, amylase, citric acid, and gluconic acid (1). Aspergillus aculeatinus is a member of the black aspergilli and closely related to Aspergillus aculeatus (2). *A. aculeatinus* has the potential for industrial application, as it produces the bioactive compound neoxaline, the antifungal compound aculeacin, and the antitumor compound paclitaxel (originally named Taxol [Bristol-Myers Squibb]) (2, 3). To date, only one *A. aculeatinus* genome has been sequenced (4).

To provide additional genomic resources for *A. aculeatinus*, we sequenced the genome of *A. aculeatinus* IC_8 after isolating it from an indoor air sample of a 23-story urban housing complex in Abidjan, Ivory Coast, that houses ∼2,000 residents. Specifically, petri dishes with Sabouraud chloramphenicol agar were left open for 24 hours and then incubated at 25°C for 3 days. We used the hyphal tipping approach followed by incubation and single spore isolation to retrieve pure culture. DNA extraction was carried out as previously described (5). Briefly, spores were plated onto potato dextrose agar (PDA) and incubated at 37°C for 96 hours. Spores were collected and directly used for DNA extraction using the MasterPure yeast DNA purification kit following the manufacturer’s instructions, with several minor modifications.

Next, 150-bp paired-end libraries were constructed and sequenced on an Illumina NovaSeq 6000 sequencer by Novogene. Raw reads were first deduplicated using Tally v15-065 with the “--with-quality” and “--pair-by-offset” options (6). Trim_Galore v0.4.2 was then used to remove residual adaptor sequences and to trim low-quality sequences using the parameters “--paired,” “--stringency 1,” “--quality 30,” and “--length 50” (http://www.bioinformatics.babraham.ac.uk/projects/trim_galore/) (7). The deduplicated and trimmed data set contained 14,017,719 paired reads with a total of 4.07 billion bp. Next, the data were error corrected, and the genome was assembled *de novo* using SPAdes v3.13.1 with the “--careful” mode and a *k*-mer range of 55, 77, and 99 (8).

The assembly consisted of 441 scaffolds, a cumulative assembly size of 36.47 Mb (nearly identical to that of the *A. aculeatinus* CBS 121060 genome [4]), an *N*_50_ value of 649,318 bp, and a GC content of 50.48%. Genome completeness was evaluated with BUSCO v3.1.0 using the “ascomycota_odb9” gene set (9). A total of 98.9% of BUSCO genes were recovered from the IC_8 genome, indicating a high-quality genome assembly.

**FIG 1:**
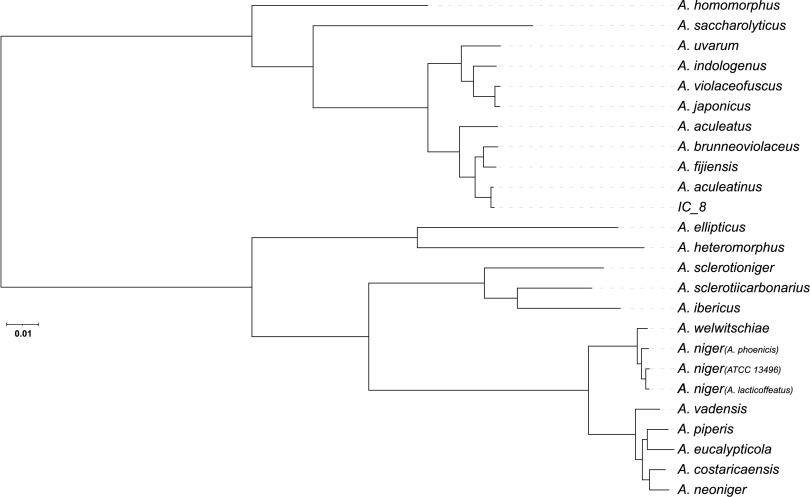
Phylogenetic relationship of 25 *Aspergillus* section *Nigri* genomes, including IC_8. The phylogeny was inferred by the approximately maximum-likelihood approach in FastTree (8) from a concatenated protein alignment of 4,680 sequences. All bootstrap branch support values were 100%. IC_8 is monophyletic with *A. aculeatinus* CBS 121060, and both taxa have short branch lengths, indicating that the species identity of IC_8 is *A. aculeatinus*. The species used are as follows (with their GenBank accession numbers for the whole-genome sequences): *A. aculeatinus* (PSTE00000000), *A. aculeatus* (GCA_001890905.1), *A. brunneoviolaceus* (PSTC00000000), *A. costaricaensis* (PSTH00000000), *A. ellipticus* (PSSY00000000), *A. eucalypticola* (MSFU00000000), *A. fijiensis* (PSTG00000000), *A. heteromorphus* (MSFL00000000), *A. homomorphus* (PSTJ00000000), *A. ibericus* (PSTI00000000), *A. indologenus* (PSTB00000000), *A. japonicus* (PSTF00000000), *A. lacticoffeatus* (MSFR00000000), *A. neoniger* (MSFP00000000), A. niger ATCC 13157 (*A. phoenicis*) (QQUR00000000), A. niger ATCC 13496 (QQZP00000000), *A. piperis* (PSTD00000000), *A. saccharolyticus* (MSFQ00000000), *A. sclerotiicarbonarius* (PSSZ00000000), *A. sclerotioniger* (MSFK00000000), *A. uvarum* (MSFT00000000), *A. vadensis* (MSFS00000000), *A. violaceofuscus* (PSTA00000000), and *A. welwitschiae* (QQZQ00000000).

To verify the species of IC_8, we conducted a phylogenetic analysis of IC_8 and 24 genomes from 22 *Aspergillus* section *Nigri* species, including *A. aculeatinus* CBS 121060 (4). For all genomes, we used the Funannotate v1.7.0 (10) pipeline to predict gene models. Next, we used Orthofinder v2.3.3 to identify orthologous genes across the 25 genomes (11). A concatenated amino acid sequence alignment was generated from 4,680 translated genes. FastTree v2.1.10 was used to infer the phylogenetic relationship of isolates from the concatenated sequence alignment, using the MLACC = 3 and nearest-neighbor interchange (NNI) options, with 100 bootstraps (12, 13). IC_8 is monophyletic with *A. aculeatinus* CBS 121060, and both taxa have short branch lengths (Fig. 1), providing clear evidence that the species identity of IC_8 is *A. aculeatinus*.

### Data availability.

The whole-genome shotgun project for *A. aculeatinus* IC_8 has been deposited in GenBank under the accession number JADPID000000000. Raw Illumina data have been deposited to the NCBI Sequence Read Archive under the BioProject accession number PRJNA675076.
